# Screening Wheat Genotypes for Specific Genes Linked to Drought Tolerance

**DOI:** 10.3390/genes15091119

**Published:** 2024-08-24

**Authors:** Ahmed Sallam, Mohamed M. H. El-Defrawy, Mona F. A. Dawood, Mostafa Hashem

**Affiliations:** 1 Genebank Department, Leibniz Institute of Plant Genetics and Crop Plant Research (IPK), 06466 Gatersleben, Germany; 2 Department of Genetics, Faculty of Agriculture, Assiut University, Assiut 71526, Egypt; defrawy@aun.edu.eg (M.M.H.E.-D.); mostafa.14224423@agr.aun.edu.eg (M.H.); 3 Department of Botany and Microbiology, Faculty of Science, Assiut University, Assiut 71516, Egypt; mo_fa87@aun.edu.eg

**Keywords:** water deficit, specific genes, breeding, *Triticum aestivum* L.

## Abstract

Drought stress, which significantly affects growth and reduces grain yield, is one of the main problems for wheat crops. Producing promising drought-tolerant wheat cultivars with high yields is one of the main targets for wheat breeders. In this study, a total of seven drought-tolerant wheat genotypes were screened for the presence of 19 specific drought tolerance genes. The genotypes were tested under normal and drought conditions for two growing seasons. Four spike traits, namely, spike length (SPL), grain number per spike (GNPS), number of spikelets per spike (NSPS), and grain yield per spike (GYPS), were scored. The results revealed that drought stress decreased the SPL, GNPS, NSPS, and GYPS, with ranges ranging from 2.14 (NSPS) to 13.92% (GNPS) and from 2.40 (NSPS) to 11.09% (GYPS) in the first and second seasons, respectively. ANOVA revealed high genetic variation among the genotypes for each trait under each treatment. According to the drought tolerance indices, Omara 007 presented the highest level of drought tolerance (average of sum ranks = 3), whereas both Giza-36 genotypes presented the lowest level of drought tolerance (average of sum ranks = 4.8) among the genotypes tested. Among the 19 genes tested, 11 were polymorphic among the selected genotypes. Omara 007 and Omara 002 presented the greatest number of specific drought tolerance genes (nine) tested in this study, whereas Sohag-5, Giza-36, and PI469072 presented the lowest number of drought tolerance genes (four). The number of different genes between each pair of genotypes was calculated. Seven different genes were found between Omara 007 and Giza-36, Omara 007 and Sohag-5, and Omara 002 and PI469072. The results of this study may help to identify the best genotypes for crossing candidate genotypes, and not merely to genetically improve drought tolerance in wheat.

## 1. Introduction

Wheat (*Triticum aestivum* L.) is one of the most important cereal crops in the world. It plays a crucial role, providing more than 20% of the calories and protein required to feed more than 35% of the world’s population across 40 countrie [1]. In terms of food security, wheat ranks as the second-most important food crop in developing countries after rice, as approximately 80 million farmers rely on its production for their livelihood [2]. Global wheat production was 783.8 million tons in 2022 [3].

The wheat crop is sensitive to heat and drought stresses, mainly at the flowering and grain development stages, which negatively impact yield and grain quality (lower 1000-grain weight and changes in protein quality) [4,5]. Drought is a global problem and will become more prevalent due to climate change.

The impact of drought on wheat varies across different stages and regions of wheat production. Generally, wheat is highly vulnerable to drought, particularly during the flowering stage [6].

During the vegetative growth stage, water scarcity significantly affects plant physiology. Drought-induced stomatal closure reduces a plant’s ability to transpire and cool itself, leading to a significant increase in leaf temperature and a decrease in photosynthetic efficiency [7]. Furthermore, drought stress during the reproductive phase of wheat, particularly during flowering and grain filling, has severe implications for grain yield. A study by Fischer et al. [8] revealed that water deficit affected pollen viability, impaired fertilization, and decreased grain set, ultimately leading to lower grain yield. In addition to physiological effects, drought stress also triggers various molecular responses in wheat. For example, the expression of stress-responsive genes and the accumulation of stress-related proteins increase under drought conditions [9].

Breeding to improve drought tolerance in wheat is an important task for plant breeders and geneticists seeking to produce drought-tolerant wheat cultivars. Phenotyping selection may be misleading because of human errors and environmental effects.

A comprehensive understanding of the genetic and molecular mechanisms governing drought tolerance in wheat is fundamental for the eventual development of drought-tolerant varieties [10]. Therefore, marker-assisted selection has been widely used in the genetic improvement of drought tolerance and in accelerating the genetic improvement of target traits. Furthermore, identifying the genes underlying drought tolerance presents a major challenge to geneticists and molecular researchers. 

Dehydration-responsive element-binding proteins (DREBs) represent a subset of specific molecular markers associated with genes responsive to drought [11]. This expansive family of DREB genes falls under the category of transcription factors (TFs) known to induce the expression of a substantial array of functional genes [12]. These functional genes play crucial roles in both the response to drought stress and the development of drought tolerance [13]. Additionally, many drought genes identified in wheat can be used to screen elite wheat genotypes. Therefore, investigating as many drought-tolerance genes in the tested wheat genotypes as possible will help in the selection of promising drought-tolerant wheat genotypes. The present study aimed to investigate yield traits in a set of seven highly drought-tolerant wheat genotypes and investigate the presence of specific drought tolerance genes in these genotypes for future molecular breeding programs to improve drought tolerance in wheat.

## 2. Materials and Methods

### 2.1. Plant Material

Seven drought-tolerant spring wheat genotypes were selected from the study of [14], in which a set of 198 spring wheat genotypes was tested under normal and drought conditions. Initially, the selection index of drought tolerance for each genotype was estimated in each growing season. The 20 drought-tolerant genotypes identified in each growing season were subsequently selected [14]. The genotypes in the tolerant group were compared between the two growing seasons [14]. As a result, seven genotypes were classified as drought tolerant in both growing seasons [14]. These seven genotypes represented three countries: Egypt (Giza-36, Omara002, GIMMIZA11, Omara007, and Sohag-5), Greece (PI469072), and Morocco (PI525241).

### 2.2. Experimental Layout

The seven genotypes were tested in two consecutive seasons, 2020/2021 and 2021/2022, under two conditions, normal (N) and drought (D), at the Experimental Field Station of the Department of Genetics, Assiut, Egypt (27°11′20.36″ N, 31°10′06.45″ E). The seven genotypes were among a tested population consisting of 198 genotypes which were evaluated by Hashem et al. [14] in two replications with randomized incomplete block design (RCBD). Each replication had 20 blocks with 10 genotypes/block. The soil at the experimental site was clay loam.

In both seasons and under both conditions, the seeds of each genotype were sown by hand in a single row measuring 1.5 m (one row/genotype). The final plant density was maintained at 15 seeds per row, with a spacing of 10 cm between seeds and 30 cm between rows. Under both conditions, the genotypes were sown on a regular wheat sowing date (20 November 2020 and 20 November 2021). Throughout the growing season, irrigation was applied every 21 days under normal conditions, with a total of seven irrigations in each growing season, whereas all the genotypes under drought conditions were irrigated twice throughout the growing season (the first irrigation was at sowing, and the second irrigation was at the booting stage). The location of the experimental field station is dry, and rainfall does not occur in this region.

### 2.3. Traits Measured

In both seasons and under each condition, ten main spikes were collected from ten plants/genotype. Four spike traits were scored for each genotype under both conditions: spike length (SPL; cm), grain number per spike (GNPS), number of spikelets per spike (NSPS), and grain yield per spike (GYPS, g). The reduction due to drought stress (RDD) for each trait in the two seasons was calculated as follows

RDD = (Xn − Xd)/Xn × 100

where Xn is the main average of the trait under normal conditions and Xd is the main average of the same trait under drought stress.

### 2.4. Selection of Genotypes Tolerant to Drought Stress

To select the best genotypes that tolerate drought stress, the seven best genotypes were identified on the basis of the iPASTIC [15] toolkit average of sum ranks (ASR) for each studied trait under each condition. The average ASR was subsequently estimated each year and over the two growing seasons for each genotype.

### 2.5. Statistical Analyses of the Phenotypic Data

The statistical analysis of the phenotypic data was performed with PLABSTAT software [16] via the following model:

*Y _ijk_ = μ + y_i_ + t_n_ +r_j_ + g_k_ + gy_ik_ + tg_nk_ + ygtr_(ijk)_ (error)* (1)

where *Yijk* is an observation of genotype *k* in year *i*, treatment *n*, replication *j*, and treatment *t*; *μ* is the general mean; *gy* is the genotype × year interaction; and *gt* is the genotype × treatment interaction. The error is year × treatment × replication × genotype interaction of genotype *k* within year *i*. Replications and years were considered random effects, whereas genotypes and treatment were considered fixed effects.

### 2.6. Molecular Screening for the Best Wheat Genotypes under Drought Stress

#### 2.6.1. DNA Extraction

The DNA was extracted from leaves of the seven drought-tolerant genotypes at the Faculty of Agriculture, Assiut University, Egypt. Genomic DNA was isolated from 6-day-old seedlings via the Thermos Scientific gene JET plant genomic DNA purification mini kit protocol, with minor modifications [17]. The quantity and quality of the purified DNA were checked via a Nanodrop spectrophotometer (Fisher Scientific, Waltham, MA, USA). Finally, concentrated DNA was used to amplify drought-specific markers via an Eppendorf thermocycler (Eppendorf SE, Hamburg, Germany). The protocols for DNA and PCR reactions were previously used under drought stress by Ahmed et al. [18].

#### 2.6.2. Drought-Specific Markers

A total of 19 specific markers were used for screening the best genotypes under drought stress. The primers for the DREB genes were obtained from Liu et al. [19]. The sequences of the other genes were obtained from EnsemblePlants (https://plants.ensembl.org/Triticum_aestivum/Info/Index), and the primers were subsequently designed via OligoPerfect Primer Designer (Thermo Fisher, Dreieich, Germany). The primer names, sequences, and corresponding annealing temperatures are presented in Supplementary Tables S1 and S2.

A 2X PCR Master Mix (Thermo Fisher, Germany) was used for the PCR experiments. The PCR tube containing the DNA sample had a total volume of 20 µL/reaction and was composed of 1.0 µL of genomic DNA, 10 µL of PCR master mixture, 0.2 µL of forward primer, 0.2 µL of reverse primer, and 8.6 µL of nuclease-free water. The reaction mixture was preheated at 94 °C for 5 min, followed by 35 cycles of 30 s denaturation at 94 °C, 30 s annealing at 52–67 °C (based on each primer’s annealing temperature), and elongation at 72 °C for 1 min.

After the last cycle, a final step was maintained at 72 °C for 5 min to allow complete extension of all amplified fragments.

#### 2.6.3. Gel Electrophoresis

The amplification products were visualized on an agarose gel in 1× TAE buffer. The agarose gel was stained with SYBR Safe DNA gel stain for 20 min. The stained agarose gel was illuminated by a gel documentation system (Ultraviolet Product, Upland, CA, USA) to assess the DNA profiles.

## 3. Results

### 3.1. Effects of Drought Stress on the Studied Traits

**Figure 1:**
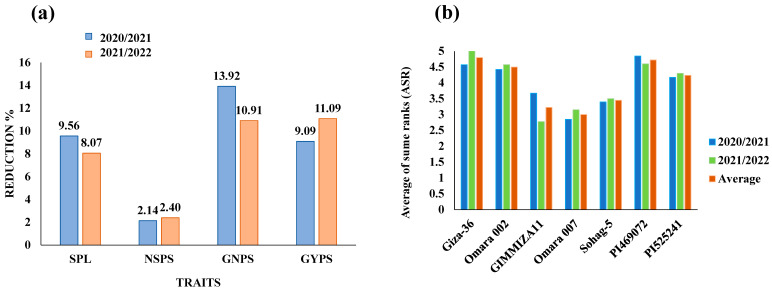
The reduction due to drought of studied traits in both seasons (**a**), and average of sum ranks (ASR) of all stress indices for each genotype in two seasons (**b**). SPL: spike length, NSPS: number of spikes per spike, GNPS: grain number per spike, GYPS: grain yield per spike.

Among the various agronomic traits, spike traits are the most important for improving wheat productivity. In the present study, drought stress decreased the scores of all the traits in the two seasons (Table 2, Figure 1a). In 2020, the GNPS had the greatest reduction due to drought stress (13.92%, from 91.31 to 78.6%), whereas the NSPS had the lowest reduction (2.14%, from 27.79 to 27.19%). In the 2021 growing season, the GYPS was the trait most strongly reduced by drought stress (from 4.7 to 4.18, 11.09%), whereas the NSPS had the lowest reduction (2.4%, from 27.79 to 27.12%).

### 3.2. Genetic Variation in Yield Traits under Drought Stress

The results of the combined analysis of variance (ANOVA) of the measured traits (Table 1) indicated that the differences among years were significant. The differences among treatments were not significant (normal vs. drought stress) for all yield traits. Highly significant genetic variation was found in all the traits among the genotypes. The G × Y interaction was significant for only the SPL and NSPS, whereas the G × T interaction was nonsignificant for all the traits.

The minimum, maximum, and mean values for all trait measurements in the two seasons for all the genotypes under normal and drought conditions are presented in Table 2. In both seasons, all the traits presented a lower average performance under drought stress than under normal conditions. The performance of each genotype for all traits under each condition and growing season is presented in Supplementary Table S3.

The phenotypic correlation for each trait between the two growing seasons under normal and drought stress conditions is presented in Table 3. A highly significant phenotypic correlation was found between the two years under each condition. Under the control, the correlation ranged from 0.72 ** (GYPS) to 0.98 ** (SPL), whereas it ranged from 0.83 ** (GNPS) to 0.99 ** (SPL) under drought stress.

The iPASTIC toolkit rank was used to determine the degree of tolerance for each genotype via various stress tolerance indices. The average ranks of the calculated indices for each genotype for each trait are presented in Table 4. In both growing seasons, the genotype with the greatest degree of drought tolerance differed by trait. The GIMMIZA 11, PI525241, and Sohag-5 genotypes were the most drought tolerant for SPL, NSPS, and GYPS, respectively. For the GYPS, Omara 007 was the most drought-tolerant cultivar in 2020/2021, and GIMMIZA 11 was the most drought-tolerant cultivar in 2021/2022. Interestingly, Omara 007 was the second-most drought-tolerant genotype SPL (in the two seasons), GNPS (in the two seasons), and GYPS (2021/2022). The average of all ranks across all traits was calculated for each genotype in each season (Figure 1b). Omara 007 was the most drought-tolerant genotype in 2020/2021, whereas GIMMIZA 11 was the most drought-tolerant genotype in 2021/2022. On average, during both seasons, Omara 007 presented the highest drought tolerance, whereas Giza-36 and PI469072 presented the lowest drought tolerance among the seven tested genotypes.

### 3.3. Gene Screening for the Drought-Tolerant Wheat Genotypes

**Figure 2:**
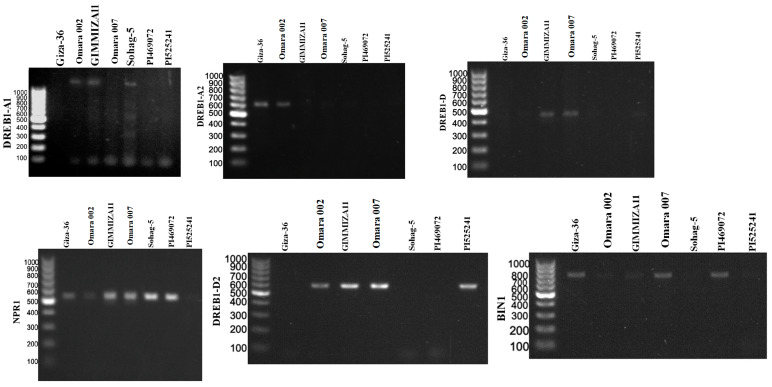

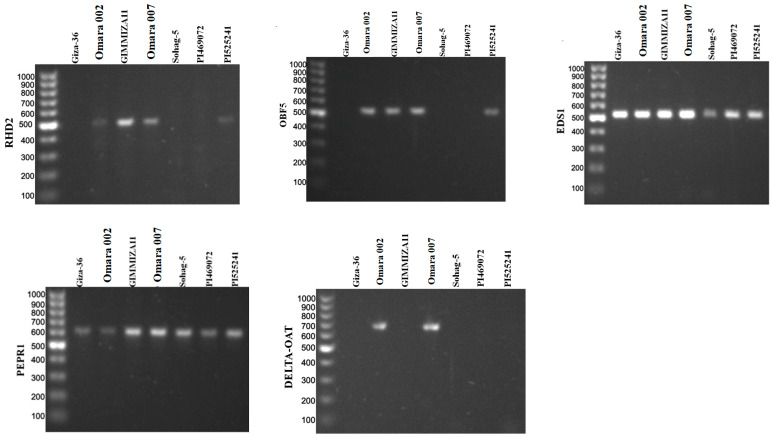
Agarose gel electrophoresis of DREB genes used in this study. Names of genotypes are supplied in Supplementary Table S1.

**Figure 3:**
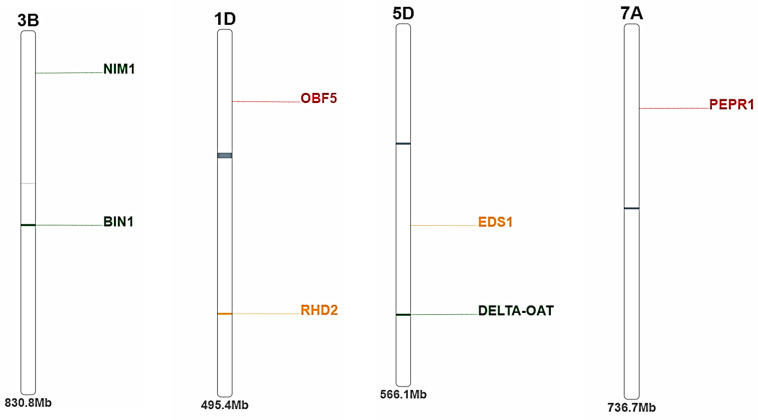
The position of the detected genes among the seven genotypes by PCR on wheat chromosomes.

The presence of 19 important drought tolerance genes was investigated in the seven drought-tolerant genotypes. Among the 19 genes, 11 were detected among the seven genotypes via PCR. The banding profile of seven wheat genotypes generated via the use of drought-specific genes is shown in Figure 2. The positions of these genes on the wheat chromosomes are presented in Figure 3. The genes were distributed in 7A (PEPR1), 1D (RHD1), 3B (NIM1 and BIN1), and 5D (EDS1 and DELTA-OAT). The protein encoded by this gene and its biological function related to drought tolerance in wheat are presented in Supplementary Table S2. All the genes were found to encode different proteins that play important roles in enhancing drought tolerance in wheat. BIN1 (3B) and PEPRI (7A) encode the protein kinase-like domain superfamily.

**Figure 4:**
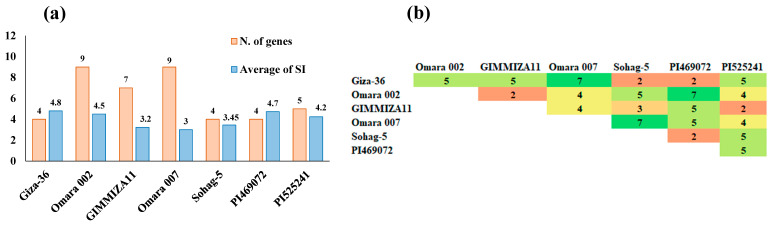
Number of genes and the average of ASR (two growing seasons) for each genotype (**a**) and the numbers of different genes found between genotypes (**b**).

Four of the DREB alleles used in this study were detected in the tolerant genotypes. High polymorphism rates were detected among the seven genotypes in the 11 tested genes. Interestingly, Omara 007 and Omara 002 presented the greatest number of specific drought genes tested in this study, with nine genes, followed by GIMMIZA11, with seven genes (Figure 4a and Table 5). On the other hand, Sohag-5, Giza-36, and PI469072 presented the lowest number of drought genes. Notably, PREP1 and EDS1 were detected in all seven genotypes (Table 5), whereas DREB1-D was detected in only one genotype, namely, Omara 007 (Egypt). PI469072 (Greece) and PI525241 (Morocco) had four and five genes, respectively. Interestingly, the most drought-tolerant genotypes, namely, Omara 007 and Omara 002, presented the greatest number of specific drought tolerance genes (nine genes) (Figure 4).

The number of different genes among each pair of genotypes was estimated (Figure 4b). The number of different genes ranged from two to seven. Notably, seven different genes were found between Omara 007 and both Giza-36 and Sohag-5.

## 4. Discussion

### 4.1. Phenotypic and Genetic Variation in Drought Tolerance

Wheat yield under drought stress suffers from severe moisture deficit throughout its growth stages, from seedling to full maturity [20]. In general, drought stress has a negative effect on physiological and agronomic traits in wheat, regardless of the genotype and duration of stress application [21]. In this study, the genetic variation in spike traits among promising drought-tolerant genotypes was studied. These genotypes were selected from a panel of 198 highly diverse wheat genotypes [14]. Analysis of variance revealed highly significant genetic variation among the drought-tolerant genotypes, indicating that these genotypes are very useful as candidate parents for crossing to produce wheat cultivars with a relatively high degree of drought tolerance. Although the average number of genotypes associated with each trait was lower under drought stress than under normal conditions, the differences between the two treatments were not significant. This result indicated that drought stress had a nonsignificant effect on spike traits compared with those under normal conditions in drought-tolerant genotypes. Moreover, these results indicated that the selection for high drought tolerance among 198 genotypes evaluated by Hashem et al. [14] was accomplished. Additionally, the nonsignificant G × T effect supported the stability of the performance of the drought-tolerant genotypes under both conditions. The high phenotypic correlations for each trait between the two years indicated that the seven genotypes presented approximately the same performance under normal and drought stress conditions. Within the whole set of genotypes (198 genotypes), there were significant differences among the treatments (drought stress and normal conditions) [14]. Additionally, NSPS was the trait least affected by drought stress, followed by SPL, GNPS, and GYPS [14], as shown among the drought-tolerant genotypes. A reduction in spike traits due to drought stress has also been reported in previous studies [22] Moreover, various factors, such as pollen abortion, an increased number of sterile tillers, and a reduction in food reserves, have the potential to negatively influence yield performance under drought stress conditions [23].

Drought stress negatively affected spike length, the number of spikelets per spike, the grain number per spike, and the grain yield per spike across all the tested genotypes. Under drought conditions, a decreasing pattern of morphological yield contributes to drought stress [24]. Drought stress led to a reduction in the grain number and number of spikelets, which ultimately caused noticeably low yield productivity. Paknejad et al. [25] reported that the improvement in cultivar yield under drought stress resulted from a longer duration of grain filling, a greater chlorophyll content, or a combination of both.

A set of nine different stress susceptibility and tolerance indices was calculated for each trait to identify the most drought-tolerant genotypes. It has been reported that the use of multiple selection indices is a more precise tool for identifying genotypes with desirable traits in breeding programs, compared to single-trait selection [26]. In this regard, Omara 007, which was the average of all ranks in each index, was the most drought-tolerant genotype. However, the overall average values for all ranks for each genotype was very close to each other, ranging from 3 (Omara 007) to 4.8 (Giza-36). The Giza-36 genotype was found to have a strong ability to extract water at very deep depths in soils [27]. Additionally, the same genotype was previously reported to have an ability to tolerate soil lead and tin contents [28]. The PI 525241 genotype was found to be a salt-tolerant genotype at the germination stage [29]. However, no previous studies have examined these sugar signaling genotypes under drought stress in wheat. Moreover, given that these seven genotypes originated from Egypt (five), Greece (one), and Morocco (one), a certain level of genetic diversity among them is anticipated. Therefore, crossing between genotypes from Egypt and Greece or Morocco will be very useful for drought-susceptible cultivars with high tolerance to drought stress. Highly divergent genotypes should be selected and crossed in breeding programs to improve cultivar crops genetically for target traits (e.g., biotic and abiotic stress tolerance [30].

### 4.2. Molecular Screening for the Tested Wheat Genotypes in This Study

Studying the specific DNA molecular markers for target characteristics in the breeding program will help improve and accelerate the breeding program. DREB genes form an extensive gene family and are categorized as transcription factors (TFs) that induce the expression of numerous functional genes [12]. A total of 210 DREB genes are associated with abiotic stress tolerance [31]. DREB genes encode dehydration-responsive element-binding proteins. The DREB gene family plays a pivotal role in drought tolerance in wheat by regulating stress-responsive genes. The activation of DREB genes by drought stress leads to the expression of stress-responsive genes that are involved in various protective mechanisms, such as osmotic adjustment, detoxification of reactive oxygen species (ROS), and maintenance of cellular structures [32]. In this study, we used DREB alleles that were previously reported by [19]. Ahmed et al. [18] used the same genes to screen drought-tolerant genotypes evaluated at the seedling stage in wheat. In this study, non-drought-tolerant genotypes presented all the DREB alleles tested. Omara 002 and Omara 007 had only three and two DREB alleles, respectively. Although the DREB gene family plays a critical role in drought tolerance, as drought tolerance is a polygenic trait controlled by many genes, it is worth investigating other important drought tolerance genes [30,33]. A set of seven drought genes were polymorphic among the drought-tolerant genotypes. The BINI and PEPRI genes encode a protein kinase-like domain superfamily. This protein plays a role in the plant response to drought stress. SnRK2 protein kinases regulate ABA signaling under drought stress. Additionally, SnRK2 was found to be associated with yield traits, especially 1000-kernel weight, in wheat [30]. The PERPI gene was found in all the genotypes, whereas BINI was found in only two. The NIM1 gene encodes an Ankyrin repeat protein, which is essential for cell growth and response to environmental stresses. High expression of this protein was found to be associated with a high survival rate under drought stress [34]. RHD2 encodes the EF-hand domain pair. The EF-hand motif was found to be included in Ca^2+^-binding proteins that are important in the plant response to drought stress and plant development during abiotic stress [35]. The DELTA-OAT gene encodes a pyridoxal phosphate-dependent transferase that is involved in plant proline biosynthesis. Additionally, these genes are highly expressed under drought stress, indicating their vital role in alleviating the effects of drought stress in wheat plants [36]. OBF 5 encodes a basic-leucine zipper (bZIP). The bZIP transcription factors are involved in various biological processes, such as flower and seed development, seed germination, light signaling, and hormone and sugar signaling [37]. Moreover, these proteins are highly expressed under abiotic stresses, such as drought stress [37]. The α/β hydrolase (EDS1 gene) was found to be highly expressed 6 h after drought stress in wheat [38].

The greatest number of gene alleles was present in both the Egyptian genotypes Omara 007 (nine genes) and Omara 002 (nine genes), whereas the lowest number (4 genes) of these genes was present in genotypes Giza-36, Sohag-5, and PI469072. Although Omara 002 had nine genes, it was ranked after Giza GIMMIZA 11, which had only four alleles. This may be because GIMMIZA 11 may contain other drought tolerance genes that were not tested in this study. In addition, some genes related to drought stress may have major or minor effects on drought tolerance. Therefore, compared with GIMMIZA 11, Omara 002 could have some minor genes. Given that these genes were, as described above, reported to be associated with drought tolerance in this study, the associations between these genes and yield traits in wheat under drought stress should be investigated. This can be investigated by genotyping a wheat population (more than 100 individuals) with these genes and phenotyping the same panel for all yield traits under drought stress. Thus, association analysis should be conducted to identify the effects (major or minor) of these genes on yield traits under drought stress.

The number of different genes among the drought-tolerant genotypes provides useful information on the diversity of drought tolerance genes. Omara 007 presented the greatest number of different genes, compared to Giza-36 and Sohag-5. Moreover, Omara 002 was found to have seven different genes relative to PI469072. It is expected that crossing these genotypes (Omara 002 and PI467072) may lead to the production of wheat cultivars with relatively high levels of drought tolerance. The cross between PI469072 and Omara 002 may be fruitful because it could not only improve drought tolerance, but also increase the genetic diversity among wheat genotypes grown in Egypt.

In conclusion, the identification and validation of novel primers for these genes are highly beneficial for wheat breeding and genetic programs. Eleven specific primers were polymorphic among the seven drought-tolerant genotypes. The high polymorphism and diversity observed among drought-tolerant genotypes suggest variation in the number of drought-specific gene haplotypes among these genotypes. The results also revealed that, compared with the Greece and Morocco genotypes, the Egyptian genotypes presented greater drought tolerance; therefore, they probably included drought genes other than those described in this study. These seven genotypes can be used for crossing in order to genetically improve drought tolerance at the adult stage in spring wheat.

## Figures and Tables

**Table 1:** Analysis of variance for scored traits under both conditions in two seasons (2020/2021 and 2021/2022).

Source	DF	SPL	NSPS	GNPS	GYPS
Y	1	21.875 **	5.59 *	1739.85 **	3.21 *
T	1	0.096	0.0182	51.07	0.23
R	1	12.99 *	2.16	2420.13 **	2.11 *
G	6	243.93 **	45.64 **	1570.55 **	3.14 **
GY	6	7.04 **	2.52 *	145.8	0.182
GT	6	1.11	1.98	120.25	0.143
GRTY	34	0.48	102.21	1.11	1.77

Year (Y), treatment (T), replication (R), and genotype (G). *, ** Significant at the 0.05 and 0.01 levels of probability, respectively. DF: degree of freedom, SPL: spike length, NSPS: number of spikelets/spike, GNPS: grain number/spike, GYPS: grain yield per spike.

**Table 2:** Minimum (Min), maximum (Max), mean, and combining correlation of each trait scored in the study under normal (N) and drought stress conditions (D) in the two seasons.

Item	Year	Normal				Drought			
		SPL	NSPS	GNPS	GYPS	SPL	NSPS	GNPS	GYPS
Minimum	2020/2021	7.67	23	69	3.07	7.67	23	56.33	3.37
	2021/2022	7.67	21.67	60	3.45	8	25	58	2.99
Maximum	2020/2021	29	31.67	119.33	6.56	20.67	30.33	100.67	5.46
	2021/2022	26.67	33.67	149	7.77	20.67	31	110.67	5.48
Mean	2020/2021	14.17	27.79	87.83	4.7	13.02	27.12	78.25	4.18
	2021/2022	14.19	27.79	91.31	4.78	12.83	27.19	78.6	4.35

SPL: spike length, NSPS: number of spikelets/spike, GNPS: grain number/spike, GYPS: grain yield per spike.

**Table 3:** Correlations between morphological traits under drought stress at the adult stages.

Control				Drought			
SPL	NSPS	NSPS	GYPS	SPL	NSPS	NSPS	GYPS
0.98 **	0.92 **	0.85 **	0.72 **	0.99 **	0.89 **	0.83 **	0.98 **

** Significant at the 0.01 level of probability. SPL: spike length, NSPS: number of spikelets/spike, GNPS: grain number/spike, GYPS: grain yield per spike.

**Table 4:** The average rank of the calculated indices for each genotype in each trait.

Season	2020/2021				2021/2022				Average
Genotype	SPL	NSPS	GNPS	GYPS	SPL	NSPS	GNPS	GYPS	
Giza-36	4	3.8	6.5	4	4.4	5.8	6.1	3.8	4.8
Omara 002	3.9	2.7	5.7	5.4	4.2	2.8	5.4	5.9	4.5
GIMMIZA11	2	4.7	3.8	4.2	2.3	4.2	3	1.6	3.225
Omara 007	3.4	4.2	2.4	1.4	3.4	3.4	2.7	3.1	3
Sohag-5	3.8	6.4	1.4	2	5.1	4.4	1.6	2.9	3.45
PI469072	5.5	4	4.5	5.4	3.8	4.4	4.8	5.4	4.725
PI525241	5.4	2	3.7	5.6	4.8	2.7	4.4	5.3	4.2375

SPL: spike length, NSPS: number of spikelets/spike, GNPS: grain number/spike, GYPS: grain yield per spike.

**Table 5:** The total number of drought genes present in each of the seven drought-tolerant genotypes.

No. of Genotypes	Genotype	Country	BIN1	NIM1	RHD2	OAT	OBF5	PEPR1	EDS1	DREB1-A1	DREB1-A2	DREB1-D	DREB1-D2	Total
1	Giza-36	Egypt	0	1	0	0	0	1	1	0	1	0	0	4
2	Omara 002	Egypt	0	1	1	1	1	1	1	1	1	0	1	9
3	GIMMIZA11	Egypt	0	1	1	0	1	1	1	1	0	0	1	7
4	Omara 007	Egypt	1	1	1	1	1	1	1	0	0	1	1	9
5	Sohag-5	Egypt	0	1	0	0	0	1	1	1	0	0	0	4
6	PI469072	Greece	1	1	0	0	0	1	1	0	0	0	0	4
7	PI525241	Morocco	0	0	1	0	1	1	1	0	0	0	1	5
	Total		2	6	4	2	4	7	7	3	2	1	4	

Where, the positive sign (1) indicates the presence of the gene, while the negative sign (0) indicates the absence of the gene or indicates that the genotype does not contain this gene.

## Data Availability

The sequence data presented in this study are not publicly available due to some ongoing projects on the same plant materials. Other data is presented in the Supplementary Files.

## Supplementary Materials

The following supporting information can be downloaded at: https://www.mdpi.com/article/10.3390/genes15091119/s1, Table S1: Primer codes, sequences of the forward and reverse primers, and annealing temperature used in this study; Table S2: List of primers used for PCR analysis and associated proteins; Table S3: The means of studied traits under normal and drought conditions in the two seasons.

## Abbreviations

N	Normal conditions
D	Drought conditions
SPL	Spike length
GNPS	Grain number per spike
NSPS	Number of spikelets per spike
GYPS	Grain yield per spike
RDD	Reduction due to drought effect
TFs	Transcription factors
RCBD	Randomized complete block design
ASR	Average of sum ranks
